# Buyang Huanwu Decoction for Healthcare: Evidence-Based Theoretical Interpretations of Treating Different Diseases with the Same Method and Target of Vascularity

**DOI:** 10.1155/2014/506783

**Published:** 2014-07-14

**Authors:** Ji-huang Li, Ai-ju Liu, Hui-qin Li, Yan Wang, Hong-Cai Shang, Guo-qing Zheng

**Affiliations:** ^1^Department of Neurology, The Second Affiliated Hospital & Yuying Children's Hospital of Wenzhou Medical University, Wenzhou, Zhejiang 325027, China; ^2^Department of Cardiology, The Second Affiliated Hospital & Yuying Children's Hospital of Wenzhou Medical University, Wenzhou, Zhejiang 325027, China; ^3^Center for Evidence-Based Medicine, Tianjin University of Traditional Chinese Medicine, Tianjin 300193, China

## Abstract

Buyang Huanwu Decoction (BHD) is a famous herbal prescription that has been used to treat stroke for centuries. Recent studies reported that the use of BHD had been extended to treat various kinds of disorders according to the TCM syndrome theory of Treating Different Diseases with the Same Method (TDDSM). Here, an overview of systematic reviews (SRs) of BHD for healthcare was conducted to interpret the TCM theory of TDDSM and its target of vascularity in an evidence-based manner. Literature searches were carried out in 5 databases to search SRs of BHD for any indication up to August 2013. Thirteen eligible SRs were identified which reported a wide range of vascular conditions. Based on the Overview Quality Assessment Questionnaire scores, the quality of included SRs was varied, with an average score of 4 points. We found that there is premature evidence for the use of BHD for healthcare, whereas BHD was well tolerable in all patients. BHD can be used to treat many disorders with the same therapeutic principle of invigorating Qi to activate blood circulation, which is essentially a manifestation of the TDDSM and is likely to account for targeting the specific pathogenesis of vascular diseases.

## 1. Introduction

ZHENG, also known as a syndrome or pattern, is the basic unit and a key concept of traditional Chinese medicine (TCM) theory that summarizes the nature, location, and pattern of diseases and has been used in China for over 3,000 years [1, 2]. Syndrome differentiation is the comprehensive analysis of clinical information gained by the four main diagnostic TCM procedures: observation, listening, questioning, and pulse analyses, which is the essential guide to treatment with TCM [3]. All diagnostic and therapeutic methods in TCM lie primarily in the syndrome differentiation [2]. Based on each individual syndrome, the precisely tailoring Chinese herbal prescription for individuals can help the improvement of efficacy of the selected TCM herbal prescription intervention [1, 3]. One example of high-quality study published in JAMA [4] indicated that using the individualized Chinese herbal medicine (CHM) for the treatment of irritable bowel syndrome is more effective than prescribing a common hypnotic prescription. Following the TCM syndrome theory, patients suffering from the different diseases might be categorized with the same syndrome (same TCM syndrome for different diseases) and may be treated by a same therapeutic approach known as Treating Different Diseases with the Same Method (Yibing Tongzhi, YBTZ) [2]. Therefore, the core of YBTZ is the TCM syndrome. Since syndrome demonstrates the specific part, cause, and property of a disease and reflects the essence of pathological changes at a stage of disease's development, a herbal prescription for YBTZ may target the specific pathogenesis of diseases. The success of personalized medicine relies on having accurate diagnostic tests that identify patients who can benefit from targeted therapies [5]. However, when the target of treatment administration is not focused but a herbal prescription so widely promulgated indications where is the target of investigation. Hence, a better understanding of the TCM theory of YBTZ and its potentially therapeutic target may contribute to evidence-based theoretical interpretations of TCM syndrome and enhance evidences of syndrome-based efficacy of CHM.

Buyang Huanwu Decoction (BHD) is a well-known classic traditional Chinese herbal prescription for stroke, which was first recorded in the* Yilin Gaicuo* (*Correction on Errors in Medical Classics*) written by Wang Qingren in 1830 during late Qing Dynasty [19]. BHD is composed of seven kinds of Chinese medicine: (A) Radix Astragali (*huang qi*), the dried roots of* Astragalus membranaceus *(Fisch.) Bge. var.* mongholicus* (Bge.) Hsiao; (B) the coda part of Radix Angelicae Sinensis root (*gui wei*), the dried lateral roots of* Angelica sinensis* (Oliv.) Diels; (C) Radix Paeoniae Rubra (*chi shao*), the dried roots of* Paeonia lactiflora* Pall.; (D) Rhizoma Chuanxiong (*chuan xiong*), the dried rhizomes of* Ligusticum chuanxiong* Hort; (E) Flos Carthami (*honghua*), the dried flowers of* Carthamus tinctorius* L.; (F) Semen Persicae (*tao ren*), the dried seeds of* Amygdalus persica* L.; and (G) Pheretima (*dilong*), the dried bodies of* Pheretima aspergillum* (E. Perrier), in the ratio of 120 : 6 : 4.5 : 3 : 3 : 3 : 3 on a dry weight basis, respectively, all of which are recorded in the* Chinese Pharmacopoeia*. Based on the TCM theory of concerted application, the main characteristic of BHD is the use of principal drug Radix Astragali in larger dose, invigorating Qi to activate blood circulation. In modern time, BHD is still widely used throughout China and elsewhere in the world for the treatment of ischemic stroke. In our group, a systematic review (SR) and meta-analysis of both randomized controlled trials and animal model experiments of focal cerebral ischemia indicated that BHD therapy appears to be able to improve neurological deficit and seems generally safe in patients with acute ischemic stroke, despite the poor methodological quality of the primary studies [9], and BHD possesses substantial neuroprotective effects in experimental stroke probably as a result of the multitarget therapy strategy typically utilized in TCM [20]. The possible protective mechanisms of BHD included improvement of hemorheology and cerebral circulation, reducing of cerebral edema and blood brain barrier permeability, reduction of excitatory neurotransmitter toxicity, reducing of Ca^2+^ overload, reduction of oxidative stress and nitration stress, anti-inflammatory effect, and antiapoptotic effect, and promotion of angiogenesis, neuronal regeneration, and synapse formation [20].

Qi deficiency and blood stasis syndrome, characterized by shortness of breath, light colored tongue, and hemorheological disorders, is one of the common TCM syndromes in the course of diseases such as cardiovascular and cerebrovascular diseases [21]. Based on the TCM theory, BHD is a classical representative prescription of Qi-tonifying and stasis-eliminating method [22]. Thus, BHD has also been extended to use for cardiovascular diseases and other disorders with Qi deficiency and blood stasis pattern according to the theory of treating different diseases with same method. One specific meta-analysis of BHD for angina pectoris with Qi deficiency and blood stasis syndrome has recently been published [18].

A SR is a literature review focused on a research question that tries to identify, appraise, select, and synthesize all high-quality research evidence relevant to that question. In the era of evidence-based medicine (EBM), SRs of several high-quality randomized controlled trials (RCTs) are crucial to EBM, which are traditionally the gold standards for judging the benefits of treatments [23]. An understanding of SRs and how to implement them in practice is becoming mandatory for all professionals involved in the delivery of healthcare. In the past few years, a wide range of SRs of BHD for a variety of diseases has been published. In addition, scientific evidence for syndrome differentiation and a breakthrough in therapeutic target of YBTZ would be beneficial for understanding the essence of syndrome. The objective of this study is thus to conduct an overview of SRs of BHD for healthcare and to explore evidence-based theoretical interpretations of YBTZ of BHD and its potentially therapeutic target.

## 2. Methods

### 2.1. Eligibility Criteria

SR is defined as an exhaustive review of the literature addressing a clearly defined question, which uses a systematic and explicit methodology to identify, select, and critically evaluate all the relevant studies and collect and analyze the data emerging from the studies included in it [24]. The reviews must include an explicit and repeatable methods section describing the search strategy and explicit inclusion/exclusion criteria involved in the effectiveness of BHD.

*Types of Reviews.* Any SRs or meta-analysis of clinical RCTs that specifically address the efficacy and safety of BHD as a primary intervention for any health condition was included. All relevant reviews were included, even if it's an empty review with no RCT identified. The review assessing multiple health conditions is excluded. Nonsystematic reviews, overviews, clinical trials, and reviews of nonclinical investigations were excluded. Reviews, which depended entirely upon previous systematic reviews for their primary data, were also excluded.

*Types of Interventions*. BHD as a monotherapy or as an adjunct therapy to conventional medicine compared with a control group receiving no intervention or other effective intervention was included. Modified BHD (BHD plus or minor few herbals) was also included, but the principal drug Radix Astragali (*huang qi*) must not be modified. SRs or meta-analysis that evaluated BHD in combination with other CHM without separately evaluating this individual prescription was excluded.

### 2.2. Literature Search

Electronic literature searches were carried out from inception to August 2013 using the PubMed, Cochrane library, China National Knowledge Infrastructure (CNKI) database, Wanfang database, and VIP database without restriction of language. The search terms used were (Bu-yang Huan-wu OR Bu-yang-Huan-wu) AND (systematic review OR meta-analysis). Chinese databases were searched using the above search terms in Chinese accordingly.

### 2.3. Study Selection and Data Collection

All articles were scanned independently by two reviewers (LJH, LAJ), and data were extracted from the articles using predefined criteria. Then, two investigators independently read the selected papers and made a final decision. Disagreements were settled by discussion or through consultation with a third party author (ZGQ).

### 2.4. Assessment Criteria of Methodological Quality

The methodological quality of all included SRs was evaluated by using the Overview Quality Assessment Questionnaire (OQAQ) that consists of 10 questions [25]. Questions 1 to 9, which were answered “yes,” “partially/cannot tell,” or “no,” address the 5 methodological aspects of SRs including search strategy, study selection, validity assessment, data analysis, and inferences. Question 10 is the overall scientific quality of the review article graded on a 7-point scale [26]. A score of 3 or less indicated extensive or major flaws, and a score of 5 or more suggested minor or minimal flaws [27].

## 3. Results

### 3.1. Description of the Screening Process

**Figure 1:**
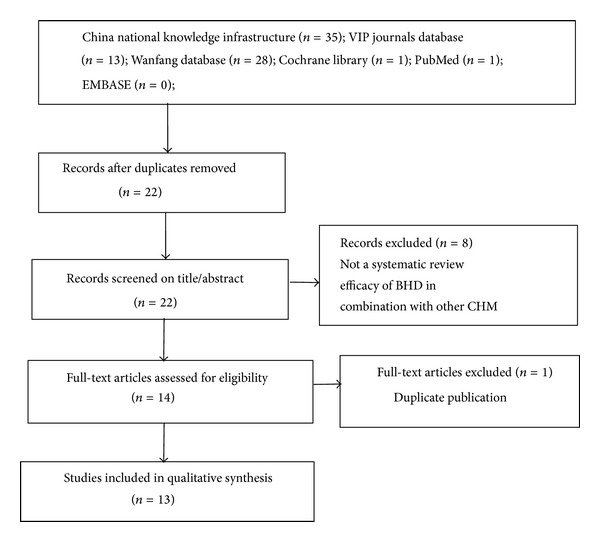
PRISMA 2009 flow diagram of the screening process.

We identified 78 potentially relevant articles. After removing duplicate articles, we were left with 22 articles. Through screening titles and abstracts, 8 were excluded because they were not SR or efficacy of BHD in combination with other CHM. After full-text evaluation on the remaining 14 articles, 1 article was excluded for duplicate publication. Finally, 13 SRs were included. The screening process is summarized in a flow diagram (Figure 1).

### 3.2. Study Characteristics

**Figure 2:**
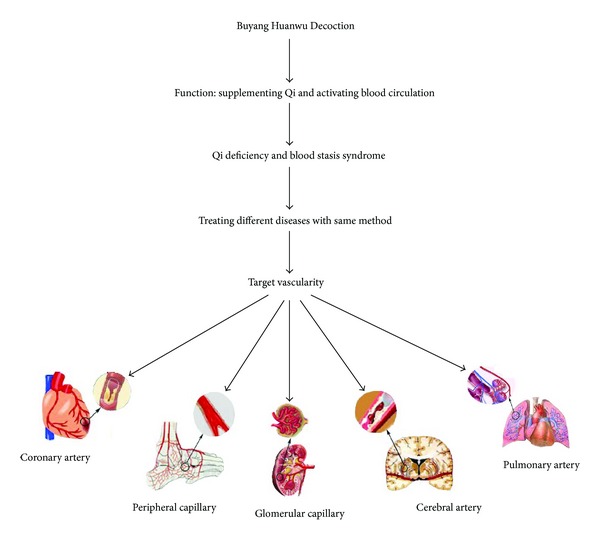
The potential target vascularity of Buyang Huanwu Decoction for different vascular diseases.

The thirteen SRs included were all conducted in China and published between 2006 and 2012. Among them, one [9] SR was published in English and 12 others [6–8, 10–16, 18] in Chinese. These SRs covered a wide range of conditions, including acute ischemic stroke (*n* = 5), acute hemorrhagic stroke (*n* = 1), chronic cor pulmonale at acute onset period (*n* = 1), primary nephrotic syndrome (*n* = 1), posterior circulation ischemia vertigo (*n* = 1), vascular dementia (*n* = 1), diabetic nephropathy (*n* = 1), diabetic peripheral neuropathy (*n* = 1), and angina pectoris of coronary heart disease with Qi deficiency and blood stasis pattern (*n* = 1). However, all these diseases targeted vascularity (Figure 2). The SRs were derived from 2 to 24 primary studies. Most of the primary studies were of poor methodological quality according to Jadad scale or Cochrane Reviewer's Handbook. All included SRs incorporated a meta-analytic approach. Key data of included SRs are summarized in Tables 1 and 2.

### 3.3. Assessing the Quality of SRs

The methodological quality of included SRs was of variable quality according to OQAQ scores. Three of them had minimal bias, that is, scoring 5 points on the OQAQ [9, 15, 18]. The other 10 SRs were mostly of poor quality [6–8, 10–14, 16, 17]. Among them, 4 SRs scored 4 points [6, 10, 16, 17], and the remaining 6 articles [7, 8, 11–14] which had major flaws scored equal to or less than 3 points. The details of the assessment of the quality of SRs are listed in Table 3.

### 3.4. Effectiveness

The conclusions of these reviews were all positive, except for vascular dementia [15] in which the total effectiveness of BHD was considered no evidence due to heterogeneity of the meta-analysis (Table 1). Firstly, BHD for cerebral vascular disease included the following: (A) acute ischemic stroke: four SRs [7–10] reported significant effects of BHD for improving the score of neurological deficit compared with control group (*P* < 0.01); one SR [10] reported improving plasma fibrinogen and the other SR [6] reported improving tumor necrosis factors and circulating endothelial cells compared with control group (*P* < 0.01); (B) acute hemorrhagic stroke: one SR [11] found that only limited evidence available for BHD was significant improving the total effective rate and the score of neurological deficit compared with control group (*P* < 0.01); (C) posterior circulation ischemia vertigo: one SR [14] reported that BHD can significantly improve the vertebral artery blood when compared with control group (*P* < 0.01); (D) vascular dementia: one SR [15] concluded that the total effectiveness rate is meaningless because of heterogeneity of the meta-analysis, although the score of minimum mental state examination (MMSE) and Hasegawa's Dementia Scale (HDS) in modified BHD group was significantly higher than that of conventional western medicine (CWM) group (*P* < 0.05). Secondly, one SR [12] evaluated BHD for chronic cor pulmonale at acute onset period, the results of which yielded a statistically significant improvement of the total effective rate with modified BHD compared with control therapy (*P* < 0.01). Thirdly, one SR [13] reported on participants with primary nephrotic syndrome. The results revealed that the total effectiveness rate of BHD plus CWM therapy group was more significantly improved than that of CWM control group (*P* < 0.01), but there was no clearly apparent beneficial effect for relapse rate (*P* > 0.01). Fourthly, one SR [16] focused on the efficacy of BHD for diabetic nephropathy. BHD plus CWM was significant improving the total effective rate and reducing urinary albumin-excretion rate, blood urea nitrogen, and 24-hour urine protein compared with CWM alone (*P* < 0.05), whereas there was no statistically significant improvement of the serum creatinine and blood glucose (*P* > 0.05). Fifthly, one SR [17] reported the efficacy of BHD in diabetic peripheral neuropathy. BHD plus CWM therapy showed a significant improvement of symptom score, nerve conduction velocity, and plasma viscosity compared with CWM plus Vitamin B therapy group (*P* < 0.05). But significant improvement of ankle-reflex was not observed (*P* > 0.05). Sixthly, one SR [18] conducted BHD for angina pectoris of coronary heart disease with Qi deficiency and blood stasis pattern. BHD plus CWM therapy significantly improved the symptom of angina pectoris and the change of electrocardiogram of angina pectoris compared with CWM control group (*P* < 0.05).

### 3.5. Adverse Drug Events

Adverse drug events monitoring was reported in detail in 2 SRs [10, 18]. One SR [10] was carried out in 2012 and only found 2 cases which suffered, respectively, from transient gastrointestinal tract reaction. In the SR by Li et al. [18], one primary study reported that there were 3 cases undergoing bellyache in the BHD group, whereas 5 cases occurred in the control group. In the remaining included SRs, nine of them described that no adverse reaction was found [6–8, 11–15, 17]. However, in the other 2 SRs [9, 16], most of primary studies did not mention whether they found adverse reaction.

## 4. Discussions

### 4.1. Summary of Main Results

To our knowledge, this is the first overview of SRs of current available evidence of BHD for healthcare. Despite the apparent positive findings, it is premature to conclude the efficacy of BHD for healthcare because the SRs themselves and their primary studies included were of generally poor quality. However, BHD is well tolerable in all patients. Another finding indicated that a remarkable multitude of SRs of BHD for a variety of diseases have been published, suggesting that the interests of the medical profession and the public in this subject have grown substantially in recent years. Impressively, BHD is commonly used in five kinds of vascular diseases according to the TCM theory of YBTZ, suggesting that BHD may target the specific pathogenesis of vascular diseases.

### 4.2. Limitations

Even though all attempts were made to interrogate and access all relevant literature, there is no absolute guarantee that all relevant articles were located in the search process. Secondly, the OQAQ scale was selected to assess various aspects of the methodological quality of SRs. However, only 3/13 scoring 5 indicate that the study has minimal flaws. Thirdly, the inherent limitations of this review are most of the primary studies included with poor methodological quality. All SRs have a tendency to publication bias within the primary research data which they include. Fourthly, all SRs originating from China potentially limited the generalizability of the findings.

### 4.3. Implication for Practice

Our overview indicated premature evidence of the effectiveness of BHD for healthcare. Moreover, BHD seems to be well tolerated in almost all included patients. We, however, noted that the absence of evidence is not the same as evidence of absence of an effect [28].

YBTZ is one of the most important characteristics in TCM theory, and as the main principle of treatment it has been widely applied in TCM clinics [29]. BHD, a well-known and canonical Chinese medicine formula, has been used for stroke for nearly 200 years. The present study indicated that BHD is commonly used in various kinds of cerebrovascular disease, including acute ischemic stroke (*n* = 5), acute hemorrhagic stroke (*n* = 1), posterior circulation ischemia vertigo (*n* = 1), and vascular dementia (*n* = 1). According to the theory YBTZ, BHD is also used for the treatment of coronary heart disease with Qi deficiency and blood stasis syndrome and improves the patients' quality of life [21]. Thus, BHD is mainly targets the vascular diseases. In other words, BHD in treatment of Qi deficiency and blood stasis syndrome is likely to aim at the common pathogenesis of vascular diseases. In the present study, BHD has been extended to other vascular and relative disorders, including chronic cor pulmonale at acute onset period, vascular complications of diabetes mellitus such as diabetic nephropathy and diabetic peripheral neuropathy and primary nephrotic syndrome, characterized by an increase in permeability of the capillary walls of the glomerulus. The theory of pattern differentiation and prescription corresponding to syndrome are two characteristic inheritance veins in TCM [30]. In modern clinics, the combination of differentiation syndrome and disease is the main therapeutic mode and feature in both TCM and integrative medicine. The present study suggested that the more suitable indications of BHD should be used in vascular disease with Qi deficiency and blood stasis syndrome.

### 4.4. Implication for Research

There are several implications for research from this overview that need to be addressed. First, methodological rigor and well-designed RCTs are urgently required in further BHD research. We suggest that some specific guidelines such as the CONSORT 2010 statement [31] guidelines and SPIRIT 2013 statement [32] guidelines for RCTs investigating CHM [33] and CONSORT for TCM [34] should be used as a combined guideline when designing and reporting RCTs for CHM. Particularly, the SPIRIT 2013 statement [32] includes a 33-item checklist plus a diagram, and each checklist item was described in detail with the rationale and supporting evidence, guidance, and model examples from actual protocols. The SPIRIT 2013 statement [32] can improve the completeness and transparency of trial protocols with providing sufficient detail to enable understanding of the background, rationale, objectives, study population, interventions, methods, statistical analyses, ethical considerations, dissemination plans, and administration of the trial; replication of key aspects of trial methods and conduct; and appraisal of the trial's scientific and ethical rigor from ethics approval to dissemination of results. High methodology quality of SRs is a prerequisite for getting confidence evidence. SRs and meta-analyses of BHD should be undertaken according to the PRISMA statement [35] and encourage the prospective registration of SRs. Second, same TCM syndrome for different diseases has its molecular bases [36]. BHD in treatment of Qi deficiency and blood stasis syndrome share the common pathogenesis of vascular diseases. Thus, to explore the molecular mechanisms of this phenomenon could contribute to scientific evidence of the theory of YBTZ and the essence of TCM syndrome. Third, the safety of TCM has become a major concern to both national health authorities and the general public now. Safety is a fundamental principle in the provision of herbal medicines and herbal products for health care and a critical component of quality control. Therefore, World Health Organization (WHO) published WHO guidelines on safety monitoring of herbal medicines in pharmacovigilance systems in 2004. In the present overview, it is difficult to draw a definite conclusion because of lack of safety data, although BHD seems well tolerable almost to all patients. Particularly, it is worth noting that BHD for acute ICH may raise a concern because promoting blood circulation for removing blood stasis may potentially increase hemorrhage, although promoting blood circulation and removing blood stasis is a common method of treatment for acute ICH patients in China according to the TCM theory of “the blood flow outside the vessels is the blood stasis.” Thus, all adverse events must be reported by the researchers participating in a clinical trial of BHD in the future.

## 5. Conclusions

Several SRs of BHD used in the treatment of a wide range of conditions have recently been published. Most of these SRs were tentatively positive. However, lack of high quality of RCTs will ultimately undermine the validity and value of the scientific evidence base. Thus, there is premature evidence of the effectiveness of BHD for healthcare. However, BHD seems to be well tolerated in almost all included patients. BHD acts to treat many disorders with the same principle, invigorating Qi to activate blood circulation. It acts upon Qi deficiency and blood stasis syndrome and is likely to aim at the common pathogenesis of vascular diseases. It thus truly encompasses the theory of YBTZ. Further study to explore the molecular mechanisms of BHD for these vascular disorders which could contribute to scientific evidence of the theory of YBTZ and the essence of TCM syndrome is needed.

## Figures and Tables

**Table 1:** Study characteristics of included systematic reviews.

Reference	Disorders	Number of randomized controlled trials	Quality assessment	Quality of randomized controlled trials	Intervention(s)	Comparison	Meta-analysis	Author's self-conclusion	Overview Quality Assessment Questionnaire OQAQ	Result	Safety
Li, 2006 [6]	Acute ischemic stroke	6	Jadad scale	Variable	BHD	Placebo or blank control	Compared total effective rate between BHD group and control group: RR 1.23, 99% CI (1.12, 1.34), *P* = 0.50. The comparison of hematocrit in BHD group and blank control group: WMD 0.40, 99% CI (−1.87, 1.07), *P* = 0.48. The comparison of whole blood viscosity in BHD group and blank control group: WMD −0.20, 99% CI (−0.76, 1.07), *P* = 0.70. The comparison of tumor necrosis factors in BHD group and blank control group: WMD −0.45, 99% CI (−0.81, −0.09), *P* = 0.001. The comparison of circulating endothelial cells in BHD group and blank control group: WMD −0.42, 99% CI (−0.78, −0.06), *P* = 0.003. The comparison of mortality rate in BHD group and blank control group: RR 0.33, 99% CI (0.01, 21.25), *P* = 0.50.	The currently available studies demonstrated that BHD is effective in patients with acute ischemic stroke but cannot lower the mortality rate.	4	+	No sufficient evidence

Li, 2006 [7]	Acute ischemic stroke	22	Jadad scale	Poor	BHD	Aspirin, placebo, or blank control	The comparison of total effective rate in BHD group and control group: RR 1.19, 99% CI (1.10, 1.30), *P* < 0.00001. The comparison of the score of neurological deficit in BHD group and blank control group: WMD −2.20, 99% CI (−3.48, −0.91), *P* < 0.0001.	BHD is effective and safe in patients with acute ischemic stroke.	3	+	Safe

Huang, 2009 [8]	Cerebral infarction	9	Jadad scale	Poor	BHD or modified BHD	Other effective therapies	The comparison of improvement of neurological deficit in experimental group and control group: OR 2.80, 95% CI (1.91, 4.09), *P* < 0.01.	BHD can improve the status of neurological deficit.	2	+	No sufficient evidence

Hao et al., 2012 [9]	Acute ischemic stroke	19	Jadad scale	Poor	BHD plus CWM or modified BHD plus CWM	CWM	Comparison of the score of neurological deficit between experimental group and control group: MD −4.65, 95% CI (−6.57, −2.27), *P* < 0.001. The comparison of effective rate of neurological deficit improvement in experimental group and control group: RR 1.18, 95% CI (1.12, 1.24), *P* < 0.001.	BHD therapy appears to be able to improve neurological deficit in patients with acute ischemic stroke and seems to be generally safe.	5	+	Safe

Liu et al., 2012 [10]	Acute ischemic stroke	10	Cochrane Reviewer's Handbook	Poor	BHD plus other effective therapies or modified BHD plus other effective therapies	Other effective therapies	The comparison of total effective rate in experimental group and control group: RR 1.23, 95% CI (1.16, 1.31), *P* < 0.0001. The comparison of fibrinogen in experimental group control group: SMD 1.98, 95% CI (0.66, 3.31), *P* = 0.003. Comparison of the score of neurological deficit between two groups: MD 5.69, 95% CI (1.59, 9.69), *P* = 0.005.	BHD is effective in patients with acute ischemic stroke. The safety of BHD is inconclusive.	4	+	No sufficient evidence

Li, 2006 [11]	Acute hemorrhagic stroke	2	Jadad scale	Poor	BHD plus CWM	CWM	The comparison of total effective rate in experimental group and control group: RR 1.35, 99% CI (1.03, 1.76), *P* = 0.005. The comparison of the score of neurological deficit in BHD groups and control groups: WMD −6.73, 99% CI (−13.71, 0.25), *P* = 0.01.	BHD is effective and safe in patients with acute hemorrhagic stroke.	3	+	Safe

Li, 2007 [12]	Chronic cor pulmonale at acute onset period	3	Jadad scale	Poor	Modified BHD	Blank control	The comparison of clinical efficacy in experimental group and control group: RR 1.18, 99% CI (1.03, 1.36), *P* = 0.002.	BHD appears to be effective and safe in patients with chronic cor pulmonale at acute onset period.	3	+	Safe

Li, 2006 [13]	Primary nephrotic syndrome	4	Jadad scale	Poor	BHD plus CWM or modified BHD plus CWM	CWM	The comparison of total effective rate in experimental group and control group: RR 1.14, 99% CI (1.01, 1.28), *P* = 0.004. The comparison of relapse rate in experimental group and control group: RR 0.62, 99% CI (0.21, 1.93), *P* = 0.27. Comparison of the incidence of adverse reaction of Cortancyl between two groups: RR 0.45, 99% CI (0.17, 1.17), *P* = 0.27.	BHD is effective and safe in patients with primary nephrotic syndrome.	3	+	Safe

Li, 2007 [14]	Posterior circulation ischemia vertigo	3	Jadad scale	Poor	BHD	Blank control	The comparison of total effective rate in experimental group and control group: RR 1.27, 99% CI (1.04, 1.54), *P* = 0.002. Comparison of the vertebral artery blood flow velocity between two groups: RR 4.50, 99% CI (2.71, 6.29), *P* < 0.00001.	BHD can promote the vertebral artery blood flow velocity and be effective and safe in patients with posterior circulation ischemia vertigo.	3	+	Safe

Shu, 2010 [15]	Vascular dementia	9	Cochrane Reviewer's Handbook	Variable	Modified BHD	CWM	The comparison of total effective rate in experimental group and control group: OR 1.17, 99% CI (1.15, 2.53), and *P* = 0.007. The comparison of improving score of MMSE of experimental group and control group: WMD 1.60, 99% CI (0.16, 3.03), *P* = 0.03. The comparison of the score of HDS of BHD in experimental group and control group: WMD 2.98, 99% CI (2.34, 3.62), *P* < 0.00001.	The statistical consequence of total effectiveness of BHD was considered meaningless due to heterogeneity of the meta-analysis. In improving score of MMSE and HDS, BHD seems more effective than western conventional medicine.	5	−	Safe

Li, 2011 [16]	Diabetic nephropathy	17	Jadad scale	Poor	BHD plus CWM	CWM	The comparison of total effective rate in experimental group and control group: OR 3.84, 95% CI (2.73, 5.42), *P* < 0.00001. Comparison of urinary albumin-excretion rate between two groups: WMD −61.76, 95% CI (−92.35, −31.16), *P* < 0.0001. Comparison of blood urea nitrogen between two groups: WMD −1.36, 95% CI (−1.70, −1.02), *P* < 0.00001. Comparison of 24-hour urine protein between two groups: SMD −0.92, 95% CI (−1.44, −0.40), *P* = 0.0005. The comparison of serum creatinine in experimental group and control group: WMD −12.82, 95% CI (−26.67, −1.02), *P* = 0.07. The comparison of blood glucose in experimental group and control group: WMD −0.33, 95% CI (−0.72, −0.06), *P* = 0.10.	The effectiveness is obviously much better in experimental group.	4	+	No sufficient evidence

Liao, 2012 [17]	Diabetic peripheral neuropathy	8	Jadad scale	Poor	CWM plus modified BHD	CWM	The comparison of total effective rate in experimental group and control group: RR 1.42, 95% CI (1.28, 1.58), *P* < 0.00001. Comparison of symptom score between two groups: WMD 1.07, 95% CI (0.81, 1.33), *P* < 0.00001. Comparison of the left sural movement nerve conduction velocity between two groups: WMD 3.79, 95% CI (2.62, 4.95), and *P* < 0.00001. Comparison of sensory nerve conduction velocity between two groups: WMD 3.97, 95% CI (2.93, 5.01), and *P* < 0.00001. The comparison of improving ankle-reflex in experimental group and control group: RR 1.30, 95% CI (0.96, 1.75), *P* = 0.09. Comparison of plasma viscosity between two groups: WMD −0.14, 95% CI (−0.23, −0.05), *P* = 0.002.	BHD is mainly effective in improving clinical symptoms and nerve conduction velocity and reducing plasma viscosity.	4	+	Safe

Li et al., 2012 [18]	Angina pectoris of coronary heart disease with Qi deficiency and blood stasis pattern	14	Jadad scale	Poor	BHD plus CWM	CWM	Comparison of clinical efficacy of improving the symptom of angina pectoris between two groups: OR 3.39, 95% CI (2.43, 4.72), *P* < 0.00001. Comparison of clinical efficacy of improving the change of electrocardiogram of angina pectoris between two groups: OR 3.27, 95% CI (1.91, 5.60), and *P* < 0.00001.	BHD can improve the symptom of angina pectoris and the change of electrocardiogram of angina pectoris but needs further study.	5	+	No sufficient evidence

Note: BHD: Buyang Huanwu Decoction; CWM: conventional western medicine; MMSE: minimum mental state examination; HDS: Hasegawa's Dementia Scale.

**Table 2:** Quality assessment and inclusion and exclusion criteria of each included systematic review.

Reference	Quality assessment	Quality of randomized controlled trials	Inclusion criteria	Exclusion criteria
Li, 2006 [6]	Jadad scale	Variable	P: patients with acute ischemic stroke within 30 days of onset and without serious organic disease and complications I: BHD C: placebo or blank control O: the mortality rate, total efficiency, cured and markedly effective rate, disability rate, improvement of neurological deficit, focal neurologic signs, rate of adverse events, complications, and laboratory test index S: RCT or quasirandomized controlled trials	N.R

Li, 2006 [7]	Jadad scale	Poor	P: patients with ischemic stroke within 30 days of onset and without serious organic disease and complications I: BHD C: aspirin, placebo, or blank control O: improvement of NIHSS score, disability rate, rate of adverse events, measurement of quality of life, the mortality rate, and measurement of activities of daily living S: RCT	(1) Nonrandomized controlled trials (2) Included patients with other diseases such as trauma, cerebral vascular malformation, intracranial aneurysms (3) Adding other medicine in the test group (4) Controlled method is cross-referenced; efficacy of medication used in control group is uncertain

Huang, 2009 [8]	Jadad scale	Poor	P: patients with acute cerebral infarction I: BHD or modified BHD C: other effective therapies O: improvement of NIHSS score, disability rate, rate of adverse events, measurement of quality of life, and the mortality rate S: RCT	(1) Nonrandomized controlled trials (2) Included patients with other diseases such as trauma, cerebral vascular malformation, and intracranial aneurysms (3) Adding other medicines in the test group (4) Studies of self-control

Hao et al., 2012 [9]	Jadad scale	Poor	P: patients of any gender, age, or ethnicity with acute ischemic stroke within 7 days of onset I: BHD and CWM or modified BHD and CWM C: CWM O: death or dependency at the end of follow-up, the neurological deficit improvement after treatment (the scores of neurological deficit improvement and the effective rate), and adverse events S: RCT	(1) Quasirandomized controlled trials or nonrandomized controlled trials (2) Studies comparing BHD therapy with another form of Chinese herbal medicine (3) Adding other Chinese herbal medicine in the treatment (4) Unclearness of the onset time (5) Not conforming to the diagnostic criteria (6) Adopting nonstandard efficacy criteria

Liu et al., 2012 [10]	Cochrane Reviewer's Handbook	Poor	P: patients of any gender and age with acute ischemic stroke within 7 days of onset I: BHD and other effective therapies or modified BHD and other effective therapies C: other effective therapies O: the neurological deficit improvement after treatment, clinical efficacy rate, and fibrinogen S: RCT	N.R

Li, 2006 [11]	Jadad scale	Poor	P: patients with hemorrhagic stroke within 30 days of onset and without serious organic disease and complications I: BHD and CWM C: CWM O: improvement of NIHSS score, disability rate, rate of adverse events, measurement of quality of life, the mortality rate, and measurement of activities of daily living S: RCT	(1) Nonrandomized controlled trials (2) Included patients with other diseases such as trauma, cerebral vascular malformation, and intracranial aneurysms (3) Adopting other medicine in the test group (4) Controlled method is cross-referenced; efficacy of medication used in control group is uncertain

Li, 2007 [12]	Jadad scale	Poor	P: patients with chronic cor pulmonale at acute onset period I: modified BHD C: blank control O: markedly effective (cardiopulmonary function improved 2 grades, symptom of cough, expectoration and asthma improved, be able to take care of oneself in daily life), effective (cardiopulmonary function improved 1 grade, symptom of cough, expectoration and asthma improved), and ineffective (cardiopulmonary function did not improve or deteriorate) S: RCT	(1) Nonrandomized controlled trials (2) Patients with acute upper gastrointestinal bleeding and disseminated intravascular coagulation (3) Controlled method is cross-referenced; efficacy of medication used in control group is uncertain

Li, 2006 [13]	Jadad scale	Poor	P: patients with primary nephrotic syndrome and without serious organic disease and complications I: BHD and CWM or modified BHD and CWM C: CWM O: complete remission (24-hour urine protein was less than 0.31 gram for more than 3 days and serum albumin was more than 35 gram per liter), partial remission (24-hour urine protein ranged from 0.31 to 2.0 gram for more than 3 days), and ineffectiveness (24-hour urine protein was more than 2.0 gram) S: RCT	(1) Nonrandomized controlled trials (2) The secondary nephrotic syndrome (3) Controlled method is cross-referenced; efficacy of medication used in control group is uncertain

Li, 2007 [14]	Jadad scale	Poor	P: patients with posterior circulation ischemia vertigo and without severe organic disease and complications I: BHD C: blank control O: transcranial Doppler (TCD), cure rate, markedly effective rate, effective rate, ineffective rate, rate of adverse events, measurement of quality of life, and measurement of activities of daily living S: RCT	(1) Nonrandomized controlled trials (2) Vertigo caused by other reasons (3) Adding other medicine in the test group (4) Controlled method is cross-referenced; efficacy of medication used in control group is uncertain

Shu, 2010 [15]	Cochrane Reviewer's Handbook	Variable	P: patients with vascular dementia and the course of treatment must be more than 2 months I: modified BHD C: CWM O: minimental state examination (MMSE), Hasegawa's Dementia Scale (HDS), and total effectiveness S: RCT	(1) Quasirandomized controlled trials or nonrandomized controlled trials

Li, 2011 [16]	Jadad scale	Poor	P: patients of any gender, age, course of disease, or race/ethnicity with diabetic nephropathy I: BHD and CWM C: CWM O: urinary albumin-excretion rate, serum creatinine, blood urea nitrogen, fasting blood glucose, effective rate, and 24-hour urine protein S: RCT	(1) Add other medicine in the test group (2) The primary studies reported unclear (3) Nephropathy caused by other diseases except diabetic (4) Patients with primary nephrotic syndrome, severe cardiovascular disease, and primary liver disease

Liao, 2012 [17]	Jadad scale	Poor	P: patients with diabetic peripheral neuropathy diagnosed according to related diagnosis standard established by Chinese Medical Association I: CWM and modified BHD C: CWM and vitamin B12/B1 O: markedly effective, effective, and ineffective S: RCT	(1) Pathological changes of central nervous system caused by diabetic or peripheral neuropathy were caused by other reasons (vasculitis, drugs, and inflammatory demyelinating neuropathy) (2) Adding other medicines in the test group (3) Case study research, experience summary, reviews, and duplicate publication (4) Animal experiment (5) Studies focus on adverse reaction of BHD

Li et al., 2012 [18]	Jadad scale	Poor	P: patients of any gender, age, or onset time with angina pectoris of coronary heart disease with Qi deficiency and blood stasis pattern I: BHD and CWM C: CWM O: improvement of symptom at the end of the treatment period, improvement of electrocardiogram, and adverse reaction S: RCT	(1) Nonrandomized controlled trials (2) Coronary heart disease does not belong to qi deficiency and blood stasis pattern (3) Not conforming to the diagnostic criteria

Note: N.R: not reported; BHD: Buyang Huanwu Decoction; RCT: randomized controlled trial; TCM: traditional Chinese medicine; CWM: conventional western medicine; NIHSS: National Institutes of Health Stroke Scale.

**Table 3:** Overview Quality Assessment Questionnaire (OQAQ) for the included systematic reviews.

Reference	(1) Were the search methods reported?	(2) Was the search comprehensive?	(3) Were the inclusion criteria reported?	(4) Was selection bias avoided?	(5) Were the validity criteria reported?	(6) Was validity assessed appropriately?	(7) Were the methods used to combine studies reported?	(8) Were the findings combined appropriately?	(9) Were the conclusions supported by the reported data?	(10) Overall score
Li, 2006 [6]	2	2	2	1	0	0	2	1	2	4
Li, 2006 [7]	1	2	2	1	0	0	2	2	2	3
Huang 2009 [8]	1	1	2	1	0	0	2	2	2	2
Hao et al., 2012 [9]	2	2	2	2	0	0	2	2	2	5
Liu et al., 2012 [10]	2	2	2	1	0	0	2	2	2	4
Li, 2006 [11]	1	2	2	1	0	0	2	2	2	3
Li, 2007 [12]	1	2	2	1	0	0	2	2	2	3
Li, 2006 [13]	1	2	2	1	0	0	2	2	2	3
Li, 2007 [14]	1	2	2	1	0	0	2	2	2	3
Shu, 2010 [15]	2	2	2	2	0	0	2	2	2	5
Li, 2011 [16]	1	2	2	2	0	0	2	2	2	4
Liao, 2012 [17]	2	1	2	2	0	0	2	2	2	4
Li et al., 2012 [18]	2	2	2	2	0	0	2	2	2	5

Note: 2: yes; 1: partly or cannot tell; 0: no.
